# Disease Characteristics, Care-Seeking Behavior, and Outcomes Associated With the Use of AYUSH-64 in COVID-19 Patients in Home Isolation in India: A Community-Based Cross-Sectional Analysis

**DOI:** 10.3389/fpubh.2022.904279

**Published:** 2022-07-06

**Authors:** Narayanam Srikanth, Adarsh Kumar, Bhogavalli Chandrasekhararao, Richa Singhal, Babita Yadav, Shruti Khanduri, Sophia Jameela, Amit Kumar Rai, Arunabh Tripathi, Rakesh Rana, Azeem Ahmad, Bhagwan Sahai Sharma, Ankit Jaiswal, Rajesh Kotecha, State Level Collaborators, Tanuja Nesari

**Affiliations:** ^1^Central Council for Research in Ayurvedic Sciences, New Delhi, India; ^2^Ministry of Ayush, Government of India, New Delhi, India

**Keywords:** Ayurveda, Ayush, AYUSH-64, COVID-19, community study, home isolation

## Abstract

**Background:**

During the second wave of the COVID-19 pandemic in India, the Ministry of Ayush conducted a community study to provide therapeutic care to patients with asymptomatic, mild, and moderate COVID-19 in home isolation based on the empirical evidence generated on the efficacy of AYUSH-64 in COVID-19.

**Objective:**

To document disease characteristics, care-seeking behavior, and outcomes in patients with asymptomatic, mild, or moderate COVID-19 in home isolation who used AYUSH-64 for COVID-19.

**Methods:**

Cross-sectional analysis of the data generated through a community study conducted in India from 08 May to 31 August 2021 was performed to study the disease characteristics, care-seeking behavior during home isolation, clinical outcomes, adverse events, and the association between various risk factors and clinical recovery during the study period. The data were collected through semi-structured questionnaires, available in electronic data collection format at the baseline, 7, 14, and 21 days. A logistic regression was performed to explore the relationship between relevant variables and clinical recovery.

**Results:**

Data from 64,642 participants were analyzed for baseline assessment, and final analysis was done for 49,770 participants. The mean age of the enrolled participants was 38.8 ± 11.7 years, and 8.4% had co-morbidities. AYUSH-64 was utilized as an add-on to the standard care by 58.3% of participants. Comparable clinical outcomes were observed in participants utilizing AYUSH-64 either as a standalone or as an add-on to standard care, in terms of clinical recovery, disease progression, the requirement for oxygen supplementation, hospitalization, ICU admission, and need for ventilator support. Younger age, having no co-morbidities or substance abuse, and having been vaccinated were associated with early clinical recovery than those who were older and not vaccinated.

**Conclusions:**

The study findings suggest that AYUSH-64 use, either standalone or as an adjunct to standard care, in asymptomatic, mild, or moderate COVID-19 is associated with good clinical outcomes. Ayush services and interventions can be effectively integrated into the mainstream public health architecture to serve public health goals.

## Introduction

The global impact of COVID-19 triggered an unprecedented and heterogeneous response from governments, and the ability of governments to act decisively and effectively was cast under public scrutiny. The timely allocation of infrastructure, human resources, and budget across different sectors and beneficiaries took a considerable risk-benefit analysis to simultaneously balance public health and economic considerations (1). Owing to the catastrophic pandemic nature and its high transmissibility, the majority of the countries targeted effective containment strategies and measures to equip themselves for effectively managing the burden of hospitalization and mortality associated with COVID-19. While quarantines, lockdowns, prophylactic medical care, and social distancing played a critical role in reducing the disease transmission, the stress on the health care system was too large, especially in countries with modest resources and health care capacity. Ensuring adequate capacity and resources to provide rapid and effective health care to the masses had a substantial economic impact on developing nations. The most effective way of handling the pandemic has been a matter of great discourse on its social, ethical, and economic aspects. It may have a potential role in policy-making decisions when it leads to community-based mass interventions. India has a rich heritage of traditional systems of medicine, including Ayurveda, Yoga, Unani, Siddha, and Homeopathy (currently regulated through the Ministry of Ayush in India), which are effectively utilized in delivering a pluralistic type of health care. The Ministry of Health and Family Welfare (MoHFW) has issued proactive guidelines from time to time in line with the global approaches and national protocol to tackle this unprecedented pandemic, and the Ministry of Ayush (MoA) has issued guidelines for the prevention and management of COVID-19 (2).

AYUSH-64 is a poly-herbal Ayurveda formulation developed by the Central Council for Research in Ayurvedic Sciences (CCRAS), MoA, Government of India, and was repurposed for the symptomatic management of COVID-19 based on the evidence generated through a clinical study on Influenza-like illness (ILI) and also a molecular docking study that revealed that phytoconstituents isolated from AYUSH-64 demonstrated anti-viral activity against SARS-CoV-2 (3, 4). The experimental studies also demonstrated immunomodulating and anti-inflammatory activities of the constituents of AYUSH-64 (5–10). Based on the clinical evidence on the therapeutic potential of AYUSH-64 in COVID-19 generated through multiple clinical trials, AYUSH-64 was positioned as a potential adjunct to standard care in COVID-19 management (11–16). It was recommended for the management of asymptomatic and mild COVID-19 in the National Clinical Management Protocol based on Ayurveda and Yoga issued by the MoA, India (2).

Pre-existing health inequalities and the burden of communicable and non-communicable diseases in India compelled the diversification of health care resources to contain COVID-19, and with the call for lockdown, health care services were prioritized for COVID-19 care. The lack of pandemic preparedness strategies resulted in all preventive and curative services, and services requiring a continuum of care, coming to a halt in the public sector (17). With the view to ensure some measure of equitable access, as well as to reduce the hospital burden in COVID-19 patients, the MoA undertook an initiative to dispense AYUSH-64 at the doorstep through Ayush health care centers in patients with asymptomatic, mild, or moderate COVID-19 who were in home isolation along with the standard care. This decentralized, participatory people-centered program was designed and executed by establishing local partnerships and networks to obtain maximum penetration within the community. The involvement of local volunteers, Ayurveda professionals, including doctors, medical students, and others, and establishing a satisfactory framework for implementation were the key highlights.

The primary objective of the study was to document disease characteristics, such as disease progression, disease severity, and clinical outcomes in asymptomatic, mild, or moderate COVID-19 patients in home isolation who used AYUSH-64. The secondary objectives of the study were to assess the care-seeking behavior (AYUSH-64 as stand-alone/or with standard care) and adverse events reported. The association between various demographic and clinical variables with clinical recovery at day 21 and the factors that may have a role in the participant's preference for using AYUSH-64 either as a standalone or as an adjunct to standard care was also included as a study objective.

## Materials and Methods

### Study Design

This is a cross-sectional analysis of data generated through a community-based distribution of Ayurvedic intervention, AYUSH-64, as standalone or as an add-on to standard care for patients patients with asymptomatic, mild or moderate COVID-19 disease in home isolation as per the guidelines issued by the MoHFW, India (18).

### Study Setting

The community-based distribution was implemented nationwide from 08 May 2021 to 31 August 2021 through 87 Ayush research and academic institutes across India.

### Study Participants

Patients with asymptomatic, mild, or moderate COVID-19, in the age group 18–60 years, with SpO_2_ levels, ≥ 94%, in home isolation as per the national guidelines, and provided consent were the study participants. The diagnosis of COVID-19 was based on a positive RT-PCR/ RAT assay or based on the presence of symptoms suggestive of clinically compatible COVID-19 illness (at least one of the following symptoms: fever, cough, difficulty in breathing, myalgia, headache, sore throat, new olfactory or taste disorder, or diarrhea) (19), or in home isolation along with any of the following criteria, i.e., those residing or working in a setting with a high risk of transmission of the virus or in an area with community transmission anytime within the 14 days before symptom onset, working in a health setting, history of contact with a probable or confirmed case, or is linked to a COVID-19 cluster.

Patients with COVID-19 in home isolation, requiring oxygen support or with SpO_2_ levels below 94%, on immunosuppressive medications, not willing to provide consent or unable to take oral medicines, and pregnant and lactating women were not included.

### Informed Consent and Ethical Consideration

The Central Ethics Committee of the CCRAS approved the study. Informed consent was obtained from all the participants who expressed willingness to participate in the study and data collection after adequate information disclosure. Due to the communicability of SARS-CoV-2, limited resources, the need to protect study personnel from infection, the methodology of door-to-door medicine distribution, and the potential for a more extensive spread through fomites such as paper, informed consent was obtained through different methods. In people using a smartphone, the image of the signed consent form was asked to be shared with the study personnel. In the areas without internet coverage or those without a smart phone the content of the study information sheet and consent were shared as an SMS, to which their consent was instructed to be sent as “I agree” or “Yes.” An image of the signed informed consent document was taken without contact from those without access to a mobile. The obtained consent was then printed and stored with other study documents or scanned into the electronic format and stored. In the case of a caregiver visiting the Ayush facility for the collection of AYUSH-64, the signature of the caregiver was obtained in the consent form. Confidentiality was maintained throughout the study, from data collection to the dissemination process.

### Framework for Implementation of the Community Study

The framework was built upon the network of Ayush institutes (both research and academic) catering to public health care services under the MoA across India. The CCRAS had designed and implemented the community study aligning with the existing advisories and guidelines issued by the MoHFW and MoA. A total of 203 Ayush professionals, including research officers, academicians, and medical officers from the 87 selected institutes, were identified as nodal officers to devise the distribution plan; establish liaisons with the directorate of health services/COVID-19 cells/ COVID care centers; provide training to non-governmental organizations (NGOs) and study personnel dispensing the medicines on procedures to be followed, systematic data collection, and data entry in electronic formats; and coordinate the daily activities. The coordinating institute also provided training on the modalities of execution and standard operating procedures (SoP) to the nodal officers. Letters seeking cooperation from stakeholders were sent to state authorities, and the nodal officers in the concerned area conducted visits initially with health authorities and local government bodies to garner cooperation and finalize the plans to enable a seamless distribution and data collection. Local NGOs were engaged with dispensing the medicine to the patients with COVID-19 in home isolation, identified through local health care directorates/ COVID-19 response cells, etc. NGOs with broad reach within the community were identified, and volunteers with enough experience and interest in working as part of the community study were contacted to volunteer. Concerned nodal officers trained them through virtual sessions to handle the medicine distribution as per the plan. During the training, the volunteers were made familiar with the methodology to be followed while dispensing AYUSH-64, including physical distancing, sanitization techniques, PPE, and other preventive measures. The medical personnel, such as doctors and nurses in the study team, were also made familiar with the selection of eligible participants and preliminary health and symptom evaluation. All the study personnel were trained on the methodology for getting informed consent, data collection, and data sharing. The baseline data was collected through face-to-face interviews, and the follow-ups on days 07, 14, and 21 were done through telephonic interviews by the study personnel at each nodal institute.

### Intervention

Patients with asymptomatic COVID-19 were advised to use two tablets of AYUSH-64 (500 mg each) twice daily with warm water after meals, while symptomatic patients (mild/moderate) were advised to use two tablets of AYUSH-64 (500 mg each) thrice daily. The intervention was advised to be used along with standard conventional care suggested by the MoHFW on clinical management of COVID-19 as per the disease severity status, assessed by the local health care provider (20). Quality-assured AYUSH-64 was procured from GMP certified manufacturer, Indian Medicines Pharmaceutical Corporation Limited (IMPCL) (Batch no.:19-APM-LDA-289; Manufacturing date: May 2021; Expiry date: April 2024). AYUSH-64 is a polyherbal formulation containing *Saptaparna* (*Alstonia scholaris* R. Br.), *Katuki (Picrorhiza kurroa* Royle ex. Benth), *Kiratatikta* (*Swertia chirata* Pexbex. Karst), and *Kuberaksha (Caesalpinia crista* L.).

### Data Sources and Data Collection Methods

The requisite information was collected through semi-structured questionnaires designed in consultation with domain experts in English language, to be filled by Ayush practitioners. Due to the community nature of the study, the questionnaire was concise, easily understandable, and structured, utilizing close-ended or dichotomous questions.

The data was collected through four different questionnaires designed for data collection at each time point as per the study design, *viz*., baseline, day 7, day 14, and day 21.

The e-version of the questionnaire finalized through iterative consultations was made available through Google forms to furnish the data. After obtaining the consent, the field personnel filled the baseline data through direct interviews. The research team at the nodal institutes filled the specific questionnaire at follow-ups through telephonic interviews at the scheduled time points. The participant reports, such as RT-PCR/RAT assay for COVID-19, other laboratory investigations, and consent, were stored electronically.

### Outcomes Measures

#### Primary Outcome

The primary outcome was to document the data on the patient characteristics, such as demographics, vaccination status (recorded as not vaccinated, fully vaccinated, and one dose vaccination done along with the name of vaccine), SARS-CoV-2 testing (whether or not testing was done, the reason for testing, testing method and date of testing), and disease characteristics (days since onset of symptoms, asymptomatic or symptomatic, if symptomatic, disease severity recorded as mild or moderate, and symptoms present). The parameters associated with disease outcomes such as the proportion of participants who attained clinical recovery (criteria of “clinical recovery” defined as normal body temperature, absence of cough or mild cough, absence of dyspnea on routine activity, absence of any other symptom/sign attributed to COVID-19, and recovery should be sustained for at least 48 h as reported by the participant), progression of the disease (from asymptomatic to symptomatic, mild/moderate disease to severe disease), the proportion of participants who achieved SARS-CoV-2 clearance defined as a single negative RAT/RT-PCR assay on day 7, 14, and 21, and the proportion of participants who required hospitalization, mechanical ventilation, oxygen supplementation, or succumbed to disease, were also assessed.

#### Secondary Outcomes

The care-seeking behavior was assessed as the utilization of AYUSH-64 as standalone (those who were not taking any standard care as per the personal preference) or add-on to standard care, and the incidence of adverse events reported was included as secondary outcome measures. The association between various risk factors and clinical recovery was also assessed as a secondary outcome measure.

### Study Size

Data was generated from 64,642 patients diagnosed with asymptomatic, mild, or moderate COVID-19 in home isolation and who participated in the community study.

### Statistical Analysis

The data obtained from the questionnaires were entered into the MS-Excel sheet and were numerically coded. This coded Excel file was then imported into STATA 16.1 (Stata Corp LLC) and used for statistical analysis. The categorical data related to the patient's demographic and disease characteristics are presented as frequency (percentage). Univariate and multivariate logistic regression was performed to explore the relationship between clinical recovery and the other demographic and clinical variables. This regression model included clinical recovery as the dependent variable, and variables like age, gender, substance abuse, co-morbidities, and vaccination status of the patients as dependent variables. The logistic regression analysis results are displayed as odds-ratio (OR) and 95% confidence intervals. A *p* < 0.05 was considered statistically significant.

## Results

### Baseline Characteristics of the Study Participants

**Figure 1:**
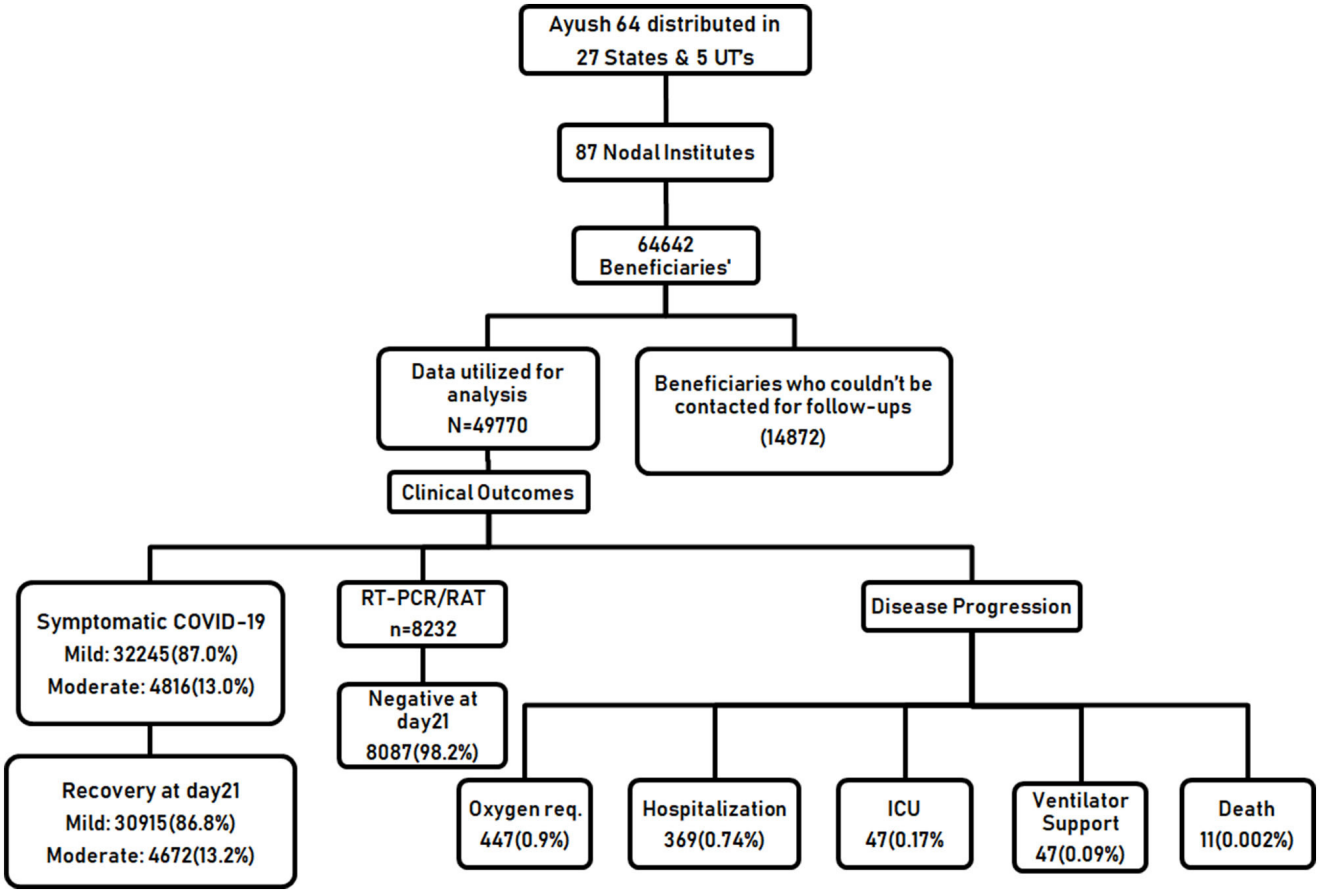
Flow diagram of the study.

The baseline characteristics of 64,642 participants in home isolation, diagnosed as having asymptomatic, mild, or moderate COVID-19, were documented and analyzed (Table 1). Data of only those participants (49,770) who were available for at least any of the scheduled follow-up visits on days 07, 14, or 21 were included in the final analysis. A total of 14,872 participants (23%) who could not be contacted at any of the scheduled follow-ups after the baseline visit were excluded from the analysis. The flow of study is given in Figure 1. The mean age of the enrolled participants was 38.8 ± 11.72 years, 37,027 (57.3%) were male and a small proportion of participants (6,560, 10.1%) had reported substance abuse. Overall, 85.9% of the study population reported to have not undergone vaccination for COVID-19. A vast majority of the participants, 49,234 (76.2%) had no history of working in the health sector/COVID-19 hospitals or other occupations that require frequent interaction with the general public and being at risk for contracting the disease. COVID-19 diagnosis in the majority of the study participants was done through a confirmatory RT-PCR test (20,943, 32.4%) or Rapid Antigen Test (20,795, 32.2%). About one-third of the participants were enrolled based on the presence of symptoms suggestive of clinically compatible COVID-19 illness. Among the participants who underwent diagnostic testing (26,787, 64.2%), reported that testing was done due to the onset of symptoms, while 10,184 (24.4%) had reported a chance of exposure with positive cases. The majority of the participants (46,208, 71.5%), were having the symptomatic disease at baseline, among which, 39,667 (85.9%), were categorized as having mild COVID-19. Overall, 5,433 (8.4%) participants reported having co-morbidities, among which diabetes mellitus and hypertension were the most common. More than half of the participants (37,674, 58.3%) used AYUSH-64 as an add-on to the standard care and 41.7% preferred to use AYUSH-64 as a stand-alone intervention for the management of COVID-19. Vitamin C (44.1%) and zinc (31.6%) were the most common supplements used, while paracetamol (43.4%) and azithromycin (26.6%) were the most common therapeutic interventions utilized.

**Table 1:** Baseline characteristics of the participants.

**Characteristics (*****n*** **=** **64,642)**		***n* (%)**
Age (in years): Mean ± SD		38.8 ± 11.72
Gender	Male	37,027 (57.3%)
Substance abuse	Present	6,560 (10.1%)
Vaccination status	Single dose/fully vaccinated	9,067 (14.1%)
Participants at higher risk of contracting the disease	General health worker/Occupation requiring frequent social/public interaction/ COVID frontline worker	15,408 (23.8%)
Co-morbidities	Present	3,644 (5.6%)
Method for diagnosis of COVID-19 disease	Positive RT-PCR/Rapid antigen assay for COVID-19	41,738 (64.6%)
	On the basis of COVID-19 like symptoms	22,904 (35.4%)
Reason for testing, (*n* = 41,738)	Chance of exposure	10,184 (24.4%)
	Onset of symptoms	26,787 (64.2%)
	Random testing (testing done in health camps, offices, stations, airports)	4767 (11.5%)
Symptomatic participants		46,208 (71.5%)
Disease severity (*n* = 46,208)	Mild	39,667 (85.9%)
	Moderate	6,541 (14.1%)
Participants taking conventional Standard Care along with AYUSH-64		37,674 (58.3%)
	Methylprednisolone	606 (0.9%)
	Dexamethasone	1,138 (1.8%)
	Inhalational Budesonide	830 (1.3%)
	Tab. Ivermectin	6,516 (10.1%)
	Tab. Paracetamol	28,052 (43.4%)
	Tab. Azithromycin	17,197 (26.6%)
	Tab. Vitamin C	28,505 (44.1%)
	Tab. Zinc	20,402 (31.6%)

### Geographical Coverage

The community study was executed in 27 States and 5 Union Territories across India through the identified 87 nodal points across the country, which indicates that the community approach was implemented as envisaged by the MoA. About one-third of the participants were enrolled from Kerala (20,419, 31.6%) and Andhra Pradesh (4,856, 7.5%) in the southern zone, followed by Maharashtra (3,704, 5.7%) in the western zone, Odisha (3,567, 5.5%) in the eastern zone, and Uttar Pradesh (3,489, 5.4%) in the central zone (Supplementary File 1).

### Clinical Recovery and Disease Severity

Among the symptomatic participants who could be contacted for follow-ups till day 21, 35,587 (96.0%) recovered completely and only 1,474 (4.0%) participants remained symptomatic. Among those who used AYUSH-64 as a standalone care, 11,846 (95.2%) reported having attained clinical recovery, while 23,741 (96.5%) underwent complete recovery among the AYUSH-64 add-on users. Among the 25.5% of the asymptomatic participants, only 1,321(10.4%) developed symptoms in the course of the study (Table 2). Cough (63.1%), fever (54.7%), fatigue/tiredness (53.9%), headache (47.8%), body ache (47.7%), and sore throat (43.1%) were the most common symptoms reported by the study participants, as depicted in Supplementary Table 1. A good proportion of participants also reported symptoms such as (24.4%), loss of taste (22.7%), rhinitis (14.5%), and loss of appetite (13.4%). Psychological symptoms such as anxiety (5.6%) and insomnia (6.7%) were reported as symptoms at baseline in a few participants. In the participants who did not undergo complete clinical recovery, the residual symptoms reported were fatigue (2.4%) and cough (1.2%).

**Table 2:** Status of RT-PCR/RAT assay and clinical recovery of participants during the study.

**Status of the participants at baseline (*n* = 49,770)**	**Status during the study duration of 20 days**	**AYUSH-64 as stand alone**	**AYUSH-64 with standard care**	**Total**
Symptomatic (n = 37061, 74.5%)	Turned asymptomatic (recovered from illness)	11,846 (95.2)	23,741 (96.5)	35,587 (96.0)
	Remain symptomatic	602 (4.8)	872 (3.5)	1,474 (4.0)
Total		12,448	24,613	37,061
Asymptomatic (n = 12709, 25.5%)	Turned symptomatic (disease progression)	686 (9.7%)	635 (11.2%)	1,321 (10.4%)
	Remain asymptomatic	6,367 (90.3%)	5,021 (88.8%)	11,388 (89.6%)
Total		7,053	5,660	12,709
Participants with negative RT-PCR assay		2,655 (98.3%)	5,432 (98.2%)	8,087 (98.2%)
Participants with positive RT-PCR assay		46 (1.7%)	99 (1.8%)	145 (1.8%)
Total^#^		2,701	5,531	8,232

*#Participants who underwent RAT / RT-PCR assay for COVID-19 again during the study period*.

### Clinical Outcomes in Terms of SARS-CoV-2 Clearance

It was observed that only 8,232 participants (16.54%) (among 49,770 utilized for analysis) had undergone a second RT-PCR/ Rapid antigen assay for COVID-19 to confirm a negative COVID-19 status. It was observed that both AYUSH-64 standalone users and add-on to standard care users demonstrate comparable outcomes (98.3 and 98.2%, respectively) in terms of attaining SARS-CoV-2 clearance (Table 2).

### Disease Progression

Only 0.90% (44) of the participants required oxygen supplementation and 0.74% (369) required hospitalization, among which 0.17% (84) required ICU admission and 0.09% (47**)** required invasive mechanical ventilator support. All the study participants who reported progression of disease depicting a possible poor outcome were followed till clinical recovery to report their disease outcomes. Among the total participants analyzed, 11 deaths (0.0002%) were reported (Supplementary Table 2).

### Incidence of Adverse Events

A total of 204 adverse events (AE) were recorded in the participants during the entire study duration, among which 171 were reported in the AYUSH-64 add-on users and 33 in the AYUSH-64 standalone users. Diarrhea was the most common AE observed (61/204), followed by gastritis (39/204), acidity (22/204), and abdominal discomfort (22/204). The AE reported in AYUSH-64 standalone users was significantly lesser than that of the add-on users. However, due to the nature of the study and the large study size, causality and relatedness could not be established. Participants on AYUSH-64 as an add-on were also on other interventions, which hindered causality evaluation.

Hypoglycemia (5/204), vertigo (4/204), and vomiting (3/204) were the AE that were reported only in the AYUSH-64 add-on users. The causality and severity of AE with AYUSH-64 use could not be established (Supplementary Table 3).

### Association of Demographic and Other Clinical Variables With Clinical Recovery

**Figure 2:**
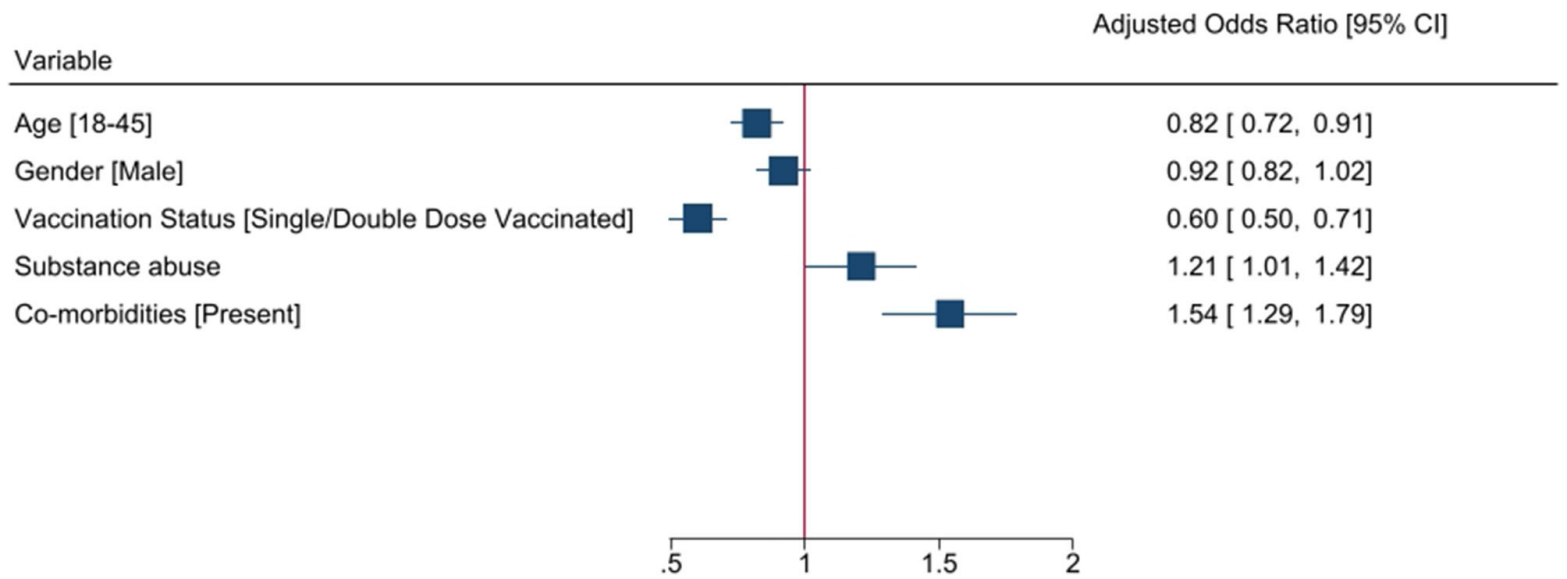
Association of baseline characteristics with clinical recovery at day 21.

Multivariate logistic regression analysis of risk factors for patients with COVID-19 to attain clinical recovery within 21 days was done using selected five variables, *viz*., age, gender, co-morbidity, substance abuse, and vaccination history, that were predictive of a possible association with the clinical outcome, which is clinical recovery within the study duration of 21 days (Table 3). In the multivariate analysis, the factors independently associated with clinical recovery in 21 days were age (OR 0.82, 95%CI 0.73–0.91), gender (OR 0.94, 95% CI, 0.85–1.05), co-morbidities (OR 1.55, 95% CI, 1.33–1.82), substance abuse (OR 1.21 95% CI 1.03–1.42), and having undergone vaccination (OR 0.68 95% CI 0.57–0.80). Younger age, being vaccinated, and having no co-morbidities or substance abuse were predictors of clinical recovery within 21 days (Figure 2).

**Table 3:** Association between demographic and clinical variables with clinical recovery.

**Variable**	**Clinically recovered**	**Remained symptomatic**	**COR (95% CI)**	***p*-value**	**AOR (95% CI)**	***p*-value**
**Age**						
18–45 years	24,603 (69.1)	954 (64.7)	0.82 (0.73–0.91)	<0.001	0.82 (0.73–0.92)	0.001
46–70 years	10,984 (30.9)	520 (35.3)	1 (Ref)		1 (Ref)	
**Gender**						
Male	20,206 (56.8)	816 (55.4)	0.94 (0.85–1.05)	0.281	0.92 (0.83–1.03)	0.133
Female	15,381 (43.2)	658 (44.6)	1 (Ref)		1 (Ref)	
**Vaccination status**						
Vaccinated	5,211(14.6)	153 (10.4)	0.68 (0.57–0.80)	<0.001	0.60 (0.51–0.72)	<0.001
Not vaccinated	30,376 (85.4)	1,321(89.6)	1 (Ref)		1 (Ref)	
**Substance abuse**						
Yes	3,529 (9.9)	173 (11.7)	1.21(1.03–1.42)	0.023	1.21 (1.02–1.43)	0.028
No	32,058 (90.1)	1,301 (88.3)	1 (Ref)		1 (Ref)	
**Co-morbidities**						
Present	3,146 (8.8)	193 (13.1)	1.55 (1.33–1.82)	<0.001	1.54 (1.31–1.81)	<0.001
Absent	32,441 (91.2)	1,281 (86.9)	1 (Ref)		1 (Ref)	

*COR, Crude Odds-ratio; AOR, adjusted odds-ratio; 95% CI, 95% confidence interval; Ref, reference category*.

### Factors Associated With Participant Preference for AYUSH-64 as an Adjunct or Standalone

Multivariate logistic regression of variables that have an association with participant's care-seeking behavior, i.e., AYUSH-64 use as standalone or as an adjunct to standard care, was done using selected six variables *viz*., age, gender, co-morbidity, vaccination history, the risk of getting infected with COVID-19, and symptomatic disease at baseline (Supplementary Table 4). In the multivariate analysis, the factors independently associated with participant preference for the use of AYUSH-64 as standalone or as an adjunct to standard care were the presence of co-morbidities such as the existence of diseases such as DM, HTN, asthma, etc. (OR 2.96, 95% CI 2.75–3.17); having undergone vaccination (OR 1.62, 95% CI 1.55–1.70); being at risk for COVID-19 (OR 1.23, 95% CI, 1.18–1.28); and symptomatic disease at baseline (OR 2.40 95% CI 2.32–2.49). Those with co-morbidities had almost three times more odds of choosing AYUSH-64 as add-on care. Likewise, those with symptomatic disease (2.40 times) and who had completed vaccination (1.62 times) were more at odds for using AYUSH-64 as an adjunct. After adjustment of confounding variables such as age, vaccination status, etc., it was that participants in the younger age group (18–45 years) were more at odds for preferring the use of AYUSH-64 as an add-on treatment.

## Discussion

In this national level community-based study, it was observed that the majority of the participants were diagnosed based on a confirmatory laboratory test (RT-PCR/RAT assay for COVID-19) and only one-third were diagnosed based on symptoms suggestive of COVID-19 compatible illness. More than half the study population was not vaccinated, and maximum participants were not among the risk groups such as the professionals, health care, and frontline workers who were in contact with the public or involved in disease surveillance or management. The care-seeking behavior observed in the study reveals that the majority of the study participants, which may depict a representative sample of the Indian population with COVID-19 in home isolation, sought AYUSH-64 as an add-on to standard care for COVID-19. Vitamin C, paracetamol, and azithromycin were the most common interventions utilized by the participants under standard care. Through this community program, Ayurveda therapeutic care was made available as a standalone therapy for 41.7% of the study population, who did not make use of standard care for the management of COVID-19 across 27 states and 5 Union Territories. A good proportion (96.0%) of the study population demonstrated clinical recovery within 21 days during the peak of the second outbreak of COVID-19, which devastated the country and overtaxed the health care facilities. Participants who used AYUSH-64 alone for COVID-19 also demonstrated comparable clinical outcomes as that of the population that utilized it as an adjunct, such as clinical recovery within 21 days, the minimal incidence of adverse events, and minimizing the chance for disease progression. A comparable response was observed in terms of the proportion of participants with SARS-CoV-2 clearance (assessed by negative RAT/ RT-PCR assay for COVID-19), irrespective of whether AYUSH-64 was used as standalone or adjunct. Based on this evidence, AYUSH-64 use is associated with good clinical outcomes in asymptomatic, mild, or moderate COVID-19.

Though the Government of India initiated the vaccination drive for eligible beneficiaries on 16 January 2021, the caseload surged during March 2021, and a gradual rise in the death rate was observed (21). Despite the extensive efforts by the government to set up COVID care centers, COVID hospitals, deploy human resources, and arrange necessary resources to contain the second outbreak of COVID-19 that officially began in April 2021 in India, 50% of COVID-19 related deaths were reported during the peak of the second outbreak between April and June 2021 (22). The risk of severity increased the rate of hospitalization and unfavorable outcomes, unavailability of hospital beds due to heavy caseload, and increased stress on health care and economic resources to cater to the needs of a sizeable ailing population prompted the MoA to distribute AYUSH-64 to patients with COVID-19 in home isolation based on the empirical evidence generated from multiple clinical studies. The timing of this initiative was critical as there were stringent restrictions such as lockdowns and curfews implemented during the study period across the country. It is worth noting that delivering door to door health care to more than 64,000 individuals in 4 months with 3 more scheduled follow-ups (day 7, day 14, and day 21) was the result of systematic end-to-end planning, implemented through effective and efficient participation of various Ayush research and academic institutes across 27 States and 5 UTs in a decentralized framework with effective collaboration with state-level local self-governance bodies, voluntary organizations, and public health care delivery systems. A large number of beneficiaries in this study also depicts the public preference for Ayurveda for therapeutic care, even in infectious diseases, which corresponds to a survey study conducted in India during the pandemic, wherein 59.6% of respondents utilized Ayurveda interventions for the management of COVID-19 and post-COVID care (23). The majority of the beneficiaries were from Kerala, Andhra Pradesh, Maharashtra, Odisha, Uttar Pradesh, and the Union Territory of Delhi, which might be attributed to the high incidence of COVID-19 and case positivity rate in these states during the study period (24).

The study findings portray that more than 98**%** of the participants who underwent the RT-PCR/RAT test for a second time attained SARS-CoV-2 clearance within 21 days, irrespective of their use of AYUSH-64 as standalone or with standard care. Among the symptomatic participants, cough, fever, headache, body ache, sore throat, fatigue, loss of smell, and loss of taste were the most common symptoms that developed during the symptomatic phase. However, fatigue, tiredness, cough, body ache, and headache were persistent as residual symptoms even after 21 days. The findings from a meta-analysis of 148 studies from nine countries regarding the clinical symptoms of COVID-19 also corroborate this observation (25). It has been observed that symptoms such as anosmia and ageusia require a prolonged recovery period in COVID-19 patients, which is also a finding in the Indian sub-continent (26). A survey study conducted in India during the COVID-19 pandemic has reported that the mean duration of clinical recovery in COVID-19 patients is 25 days, with no difference in the recovery time between males and females, and in patients older than 60 years and younger (27). The findings in this community study reveal that symptoms such as cough and fatigue persist for more than 21 days in a very small proportion of the participants despite the type of health care utilized.

Only a small proportion of participants reported worsening/progression of disease in terms of hospitalization (0.74%), oxygen supplementation (0.90%), the requirement for ICU care (0.17%), or mechanical ventilator (0.09%) support in this study, while 20–23% of the active cases needed hospital care during the second outbreak of COVID-19 in India (28). Death as a complication of COVID-19 was observed in a minimal number of participants in the study. This may be considered a good outcome, and coupled with the evidence from the previous clinical studies in which AYUSH-64 as an adjunct to conventional standard care demonstrated better clinical recovery with no disease progression compared to standard care alone in COVID-19 (11–16), it is possible to say that AYUSH-64 use in COVID-19 may correlate with better clinical outcomes even if used alone. Adverse events reported in this community study were very minimal, and none of the events required the need for medical consultation or hospitalization. The study findings suggest that using AYUSH-64 for the management of COVID-19 patients in home isolation is no cause for concern regarding safety and tolerability. Having been vaccinated or being younger was associated with better clinical outcomes in terms of clinical recovery within 21 days. The odds of those who did not undergo clinical recovery within 21 days were more among those with co-morbidities and substance abuse issues. The factors that guide a patient's preference for using Ayurveda interventions as an adjunct to standard care for the management of COVID-19 were as follows: being at risk for COVID-19, having undergone vaccination, symptomatic disease, and presence of co-morbidities. The outcomes of this study reveal that the Indian population adopts Ayush interventions for the management of infectious diseases, and the preference for using standalone Ayurveda care might be attributed to the fact that the faith and reliance on Ayush systems are firmly rooted in the Indian heritage, popularity, and previous experiences in terms of utility, accessibility, and flexibility. The utilization of complementary and alternative medicine (CAM) services around the globe are reported to be between 9% and 65%. The use of CAM in India amounts to 65%, while in Asia, it is observed to be around 80% (29). The results of an observational study conducted during COVID-19 convey that a considerably large number of the Indian population utilized Ayush-based measures for COVID-19 prevention, as is observed in the care-seeking behavior of the study participants (30).

The interim guidance published by the WHO on 7 March 2020, addressing the community spread of COVID-19, opined that the prevention of COVID-19 would be possible through the development of coordination mechanisms not just in health but also in other areas which encompass the entirety of society, and keeping this in view, community participatory mechanism of engaging volunteers and dispensing medicines was sought to help the government in reducing the health burden attributed to COVID-19 (31).

An inclusive integrative health approach, structured with an operational component to create and mobilize an operational workforce and expertise to serve societal and public health goals, is the best solution to tackle such illnesses of a pandemic nature. The Ayush knowledge, practices, and human resources functioning outside the mainstream health architecture can be well integrated through a participatory approach in collaboration with public health providers for better outcomes.

## Limitations

Due to the stringent restrictions laid down during the pandemic, mobilization of human resources to conduct follow-ups through direct assessments was not possible, and follow-ups were done through telephonic communication. A good proportion of the study participants could not be contacted through telephone during all the four scheduled follow-ups, and so were not utilized for analysis. The clinical outcomes in terms of negative RT-PCR/RAT assay for COVID-19 could only be assessed in a small proportion of the total study participants, as a second test was not mandated as per the existing government guidelines.

## Conclusions

The outcomes of this community-based interventional study highlight that a significant proportion of the public residing across diverse demographics opted to use Ayurveda intervention (AYUSH-64) as standalone or adjunct to standard conventional care in managing COVID-19. The use of AYUSH-64 is associated with good clinical outcomes in patients with asymptomatic, mild, or moderate COVID-19 in home isolation. A decentralized and participatory community approach can effectively use the existing public health machinery to deliver integrated care services, utilizing the beneficial effects of Ayurveda during the pandemic.

## Data Availability Statement

The original contributions presented in the study are included in the article/Supplementary Material, further inquiries can be directed to the corresponding author/s.

## Ethics Statement

The studies involving human participants were reviewed and approved by Central Ethics Committee, CCRAS. The patients/participants provided their written informed consent to participate in this study.

## State Level Collaborators

AIIA Delhi: Tanuja Nesari, Mahesh Vyas, Umesh Tagade; ITRA Jamnagar: Anup Thakar; Nilesh Bhatt, Kalpesh Dattani, Sagar Bhinde; NIA Jaipur: Sanjeev Sharma; Pawan Kumar Godatwar, Nisha Ojha, H.M.L.Meena, Harish Bhakuni; NEIAH Shillong: Pradeep Kumar Goswami, Bishnu Choudhury; RARI Vijayawada: Nishanth K, AJV Sai Prasad, Sujata Dhoke, Midhuna Mohan K, Savita Gopod; RARI Itanagar: Arvind Kumar; CARI Guwahati: Ekta Dogra, G.K.Bora, Pravin K S, Pravin Masarom Radheshyam, P.L.Bharati, Jeuti Rani Das; RARI Patna: Vimal Tewari, Deepika Tewari, Ritika Mishra, Kuldeep, D.S.Rotwar; RARI Ahmedabad: Anil Ahvad, Sumed Paikrao; CARI Delhi: Amit Madan, Nandini Jadhav; RARI Mandi: Vikas Nariyal, Kavita Vyas, Anubha Chandla, Vineeta Negi, Chris Antony; RARI Jammu: Vipin Sharma, Poonam Mohod, Subhash Sharma, Meenakshi Suri, Aaditya Shah; CARI Bengaluru: G.V.Ramana, Tejaswini C, Raghavendra, S.K.Giri, Shashidhar Doddamani, Shubhashree M.N, Srinibash Sahoo; NARIP Cheruthuruthy: K.M.Pratap Shankar, Parvathy.G.Nair, Devi R Nair, Krishna Kumar V., P.P. Pradeep Kumar, Remya E; RARI Thiruvananthapuram: Karthika A P, Sinimol. T.P, Meghna P P, Praveen Balakrishnan, Emy.S.Surendran, Varsha Sumedhan; RARI Gwalior: Amit Kumar, S.B.Singh, Neelam Singh, Anil Mangal, Deepa Sharma; CARI Mumbai: Laxman Bhurke, Dattatray Dighe, Kuldeep Choudhary, Saylee Deshmukh, Sneha Marlewar, Shyam Kale; RARI Nagpur: U.R.Shekhar Namboori, Savita Sharma, Priya Thakre, Prashant Shinde, Balaji Potbare; RARC Nagaland: Deepak Rahangdale, Gwachung Magh; CARI Bhubaneswar: G.C.Bhuyan, P.Panda, K.K.Ratha, Krishna Rao, Indu S, A.K.Panda, Banamali Das, Susmita Ota; CARI Patiala: Rinku Tomar, Harbans Singh, Sandeep Baheti, Sanjeev Kumar, Mahesh S, Sangeeta Sangvikar; RARI Jaipur: S.K.Vedi, Swati Sharma, V.B.Kumawat, Suhash Choudhary, Monika Kumari, Indu P P; RARI Gangtok: Rahul D.Ghuse, Shriprakash, Shrawan Kumar Sahu, Ashok Kumar Sinha; RARI Chennai: P.Srinivas, K.Prameela Devi, Asha S; RARC Tripura: Sojeetra Niral; RARI Lucknow: Karisma Singh, Kamble Pallavi, Ravi Ranjan Singh, Anjali B Prasad, Mayur Surana, Sanjay Kumar Singh, Harit Kumari, A.K.Srivastava; RARI Ranikhet: Tarun Kumar, Deepshikha Arya; CARI Kolkata: D.S.Sahu, Tushar Kanti Mondal, L.D.Barik, Suparna Saha, Ranjita Ekka, Shakti Bhushan, Achintya Mitra, Saroj Kumar Debnath, Debajyoti Das; RARI Port blair: Akashlal M, Abhayadev A; RARCMMMR Goa: Hemant Gupta, Ajay P Yadav; CCRUM: Asim Ali Khan, NRIUMSD Hyderabad: Munawar H Kazmi, Minhaj; RRIUM Delhi: Rahat Raza; CRIUM Lucknow: Md.Nafees Khan; RRIUM Patna: Md. Ishtiyaq Alam; RRIUM Mumbai: Haseeb Alam Lari; RRIUM Chennai: N. Zaheer Ahmed; RRIUM Bhadrak: Hakimuddin Khan; RRIUM Kolkata: Younis Iftikhar; RRIUM Srinagar: Seema Akbar; RRIUM Aligarh: Sheeren Afza; HAKILHRUM Delhi: Mohammad Fazil; RRC Allahabad: Ashok Kumar; CRU Meerut: Mohd Tarique; CRU Burhanpur: Amir Faisal Khan; CRU Bhopal: Amir Faisal Khan; CRU Kerala- Aijaz Ahmed; CCRH: Anil Khurana; NHRIMH Kottayam (Kerala): S. Karunakara Moorthi; CRI Noida: Subhash Kaushik; CRI Jaipur- Nitin Kumar Saklani; RRI Mumbai: B. S. Rawat; RRI Gudivada: Brunda Bezawada; RRI Shimla: Sunil Ramteke; RRI Puri: A.K. Prusty; RRI Guwahati: Liyi Karso; RRI Manipur: Amit Srivastav; RRI Tripura: Ratan Chandra Shil; RRI Kolkata: Partha Pratim Pal; HDRI Lucknow: Lipipushpa Debata; CRU Tirupati: G. Ravi Chandra Reddy; CRU Ranchi: Sunil Prasad; CRU Port blair: Uttam Singh; CRU Darjeeling: Baidurjya Bhattacharjee; CRU Gangtok: Santosh Kumar Tamang; CRU Puducherry: Ravi kumar Sadarla; CRU Mizoram: Pawan Sharma; Homeopathic Medical College Bhubaneswar: Amulya Ratna Sahoo; CVU Patna: Vibha; DSU Hyderabad: P Prasad; HRID Chennai: D. Karthikeyan; CCRYN: Raghvendra Rao; PGIYNER Harayana: Surender Sandhu; CCRYN Rohini (Delhi): Mohan Rao; PGIYNER Karnataka: Vadiraj HS; MDNIY Delhi: Ishwar V. Basavaraddi, Ishwar N Achary; NIN Pune: K Satyalakshmi, Shivkesh, P.Yuvaraj Paul; NIH Kolkata: Subhas Singh, Austin Jose; NEIFM Arunachal Pradesh: Robindra Teron, Imlikumba; NIUM Bangalore: Addul Wadud, Abdul Nasir Ansari, Tariq Nadeem Khan, Abdul Moheen; NISR Leh: Tsewang Dolma, Tenzin Tenba; RAV Delhi: Anupam Srivastav, N. Ramakrishnan; Directorate of Ayush, Gujarat: Surendra Soni, Ram Shukla, Rohini Salve, M.N. Shaikh, Daxen Trivedi, Shital Bhagiya, Asha Patel, Anup Indoriya, Rachna Gandhi, Naresh Jain, Nirmal Chavada, Rahul Shingadiya, Nilesh Bhadraka, Nrupesh Gupta, Dilip Italiya, Piyush Shah, Maya Chaudhari, Sumit Patel, Bhavin Chaudhari, Mehul Parmar.

## Author Contributions

NS: conceptualization, methodology, questionnaire development, project administration, resources, and validation. AK: methodology, project administration, co-ordination, and validation. BC, BY, and SK: methodology, questionnaire development, and writing—review and editing. RS: questionnaire development, data curation, formal analysis, and writing—review and editing. SJ: methodology, questionnaire development, visualization, and writing—review and editing. AR: visualization and writing—original draft. AT: data curation and formal analysis. RR: questionnaire development, data curation, and formal analysis. AA: visualization and writing—review and editing. BS: resources and writing—review and editing. AJ: data curation. RK: conceptualization, project administration, resources, and supervision. State Level Collaborators: investigation. All authors read, provided feedback, and approved the final version of the manuscript.

## Funding

The program was implemented through the Research Councils and National Institutes under the MoA, Government of India.

## Conflict of Interest

The authors declare that the research was conducted in the absence of any commercial or financial relationships that could be construed as a potential conflict of interest.

## Publisher's Note

All claims expressed in this article are solely those of the authors and do not necessarily represent those of their affiliated organizations, or those of the publisher, the editors and the reviewers. Any product that may be evaluated in this article, or claim that may be made by its manufacturer, is not guaranteed or endorsed by the publisher.
