# Supplementary material for: Disease Characteristics, Care-Seeking Behavior, and Outcomes Associated With the Use of AYUSH-64 in COVID-19 Patients in Home Isolation in India: A Community-Based Cross-Sectional Analysis
Source: Front Public Health. 2022 Jul 6;10:904279. doi: 10.3389/fpubh.2022.904279 (PMC9310753; doi:10.3389/fpubh.2022.904279)
Supplement: Supplementary file 1 [file Table_1.DOC]

**Table 1: Clinical presentation of the participants reported in the study**

| **Symptoms** | **Present at** | **AYUSH-64 as stand alone**  **(n = 17165)** | **AYUSH-64 as adjunct with standard care**  **(n=19896)** | **Total (n=37061)** |
| --- | --- | --- | --- | --- |
| **Fever** | **Baseline** | 5966 (34.8%) | 14297 (71.9%) | 20263 (54.7%) |
|  | **Final Assessment** | 76 (0.4) | 93(0.5) | 169(0.5) |
| **Sore throat** | **Baseline** | 5438 (31.7%) | 10522 (52.9%) | 15960 (43.1%) |
|  | **Final Assessment** | 71(0.4) | 145(0.7) | 216(0.6) |
| **Cough** | **Baseline** | 7609 (44.3%) | 15778 (79.3%) | 23387 (63.1%) |
|  | **Final Assessment** | 172(1.0) | 265(1.3) | 437(1.2) |
| **Headache** | **Baseline** | 5634 (32.8%) | 12090 (60.8%) | 17724 (47.8%) |
|  | **Final Assessment** | 54(0.3) | 135(0.7) | 189(0.5) |
| **Body ache** | **Baseline** | 5538 (32.3%) | 12158 (61.1%) | 17696 (47.7%) |
|  | **Final Assessment** | 98(0.6) | 203(1.0) | 301(0.8) |
| **Fatigue/Tiredness** | **Baseline** | 6309 (36.8%) | 13649 (68.6%) | 19958 (53.9%) |
|  | **Final Assessment** | 339(2.0) | 566(2.8) | 905(2.4) |
| **Difficulty in breathing** | **Baseline** | 979 (5.7%) | 1782 (9.0%) | 2761 (7.4%) |
|  | **Final Assessment** | 23(0.1) | 46(0.2) | 69(0.2) |
| **Loss of smell** | **Baseline** | 2420 (14.1%) | 6614 (33.2%) | 9034 (24.4%) |
|  | **Final Assessment** | 13(0.1) | 56(0.3) | 69(0.2) |
| **Loss of taste** | **Baseline** | 2501 (14.6%) | 5925 (29.8%) | 8426 (22.7%) |
|  | **Final Assessment** | 14(0.1) | 40(0.2) | 54(0.2) |
| **Rhinitis** | **Baseline** | 1856 (10.8%) | 3532 (17.8%) | 5388 (14.5%) |
|  | **Final Assessment** | 10(0.1) | 24(0.1) | 34(0.1) |
| **Insomnia** | **Baseline** | 610 (3.6%) | 1879 (9.4%) | 2489 (6.7%) |
|  | **Final Assessment** | 19(0.1) | 60(0.3) | 79(0.2) |
| **Anxiety** | **Baseline** | 634 (3.7%) | 1423 (7.2%) | 2057 (5.6%) |
|  | **Final Assessment** | 18(0.1) | 52(0.3) | 70(0.2) |
| **Diarrhea** | **Baseline** | 368 (2.1%) | 1050 (5.3%) | 1418 (3.8%) |
|  | **Final Assessment** | 19(0.1) | 14(0.1) | 33(0.1) |
| **Vomiting** | **Baseline** | 356 (2.1%) | 782 (3.9%) | 1138 (3.1%) |
|  | **Final Assessment** | 42(0.3) | 17(0.1) | 59(0.2) |
| **Loss of appetite** | **Baseline** | 1178 (6.9%) | 3790 (19.0%) | 4968 (13.4%) |
|  | **Final Assessment** | 78(0.5) | 49(0.3) | 127(0.3) |
| **Pain Abdomen** | **Baseline** | 646 (3.8%) | 737 (3.7%) | 1383 (3.7%) |
|  | **Final Assessment** | 138 (0.8) | 48 (0.3) | 186 (0.5) |

**Table 2**: Clinical outcomes in terms of disease progression and severity

| **Parameters of disease progression/ severity** | **AYUSH-64 as standalone**  **(n = 19501)** | **AYUSH-64 as adjunct with standard care**  **(n = 30269)** | **Total**  **(n = 49770)** |
| --- | --- | --- | --- |
| **Oxygen requirement** | 115 (0.58%) | 332 (1.09%) | 447 (0.90%) |
| **Hospitalization** | 76 (0.38%) | 293 (0.96%) | 369 (0.74%) |
| **ICU setting** | 13 (0.06%) | 71 (0.23%) | 84 (0.17%) |
| **Mechanical Ventilator support** | 11 (0.05%) | 36 (0.11%) | 47 (0.09%) |
| **Death in hospital** | 02 | 07 | 09 |
| **Death at home** | 01 | 01 | 02 |

**Table 3**: Incidence of adverse events during the study

| **Adverse Events** | **AYUSH-64 as standalone**  **(n = 19501)** | **AYUSH-64 as adjunct with standard care**  **(n = 30269)** | **Total**  **(n = 49770)** |
| --- | --- | --- | --- |
| Abdominal discomfort | 01 | 21 (0.07%) | 22 (0.04%) |
| Abdominal pain | 03 | 11 (0.04%) | 14 (0.03%) |
| Acidity | 03 | 19 (0.06%) | 22 (0.04%) |
| Allergic rhinitis | - | 02 | 02 |
| Diarrhoea | 07 | 54 (0.18%) | 61 (0.12%) |
| Gastritis | 08 | 31 (0.10%) | 39 (0.08%) |
| Hypoglycemia | - | 05 | 05 |
| Itching | 06 | 13 (0.04%) | 19 (0.04%) |
| Skin rashes | 04 | 08 | 12 (0.02%) |
| Vertigo | - | 04 | 04 |
| Vomiting | - | 03 | 03 |
| Burning micturition | 01 | - | 01 |
| **Total AEs reported** | 33 | 171 | 204 |

**Table 4: Factors that have an association with the participant preference for AYUSH-64 as stand-alone or add-on with standard care**

| **Variable** | **Category** | **AYUSH-64 as stand-alone (n = 26965)** | **AYUSH-64 as adjunct with standard care**  **(n = 37677)** | **COR (95%CI)** | | **AOR (95%CI)** | |
| --- | --- | --- | --- | --- | --- | --- | --- |
| **Gender** | Male | 15552 (57.7) | 21475 (57.0) | 0.97 (0.94-1.00) | | 0.96 (0.93-0.99) | |
|  | Female | 11413 (42.3) | 16202 (43.0) | *Ref* | | | |
| **Age** | 18-45 | 18766 (69.6) | 25914 (68.8) | 0.96 (0.93-1.00) | | 1.18 (1.14-1.23) | |
|  | 46-70 | 8199 (30.4) | 11763 (31.2) | *Ref* | | | |
| **Co-morbidities** | Yes | 1126 (4.2) | 4307 (11.4) | 2.96 (2.75-3.17) | 2.95 (2.75-3.16) | | |
|  | No | 25839 (95.8) | 33370 (88.6) | *Ref* | | | |
| **Vaccination Status** | Vaccinated | 2900 (10.8) | 6167 (16.4) | 1.62 (1.55-1.70) | | 1.56 (1.49-1.64) | |
|  | Not Vaccinated | 24065 (89.2) | 31510 (83.6) | *Ref* | | | |
| **At Risk for COVID-19** | Yes | 5846 (21.7) | 9562 (25.4) | 1.23 (1.18-1.28) | | 1.22 (1.18-1.27) | |
|  | No | 21119 (78.3) | 28115 (74.6) | *Ref* | | | |
| **Symptomatic at baseline** | Yes | 16448 (61.0) | 29760 (79.0) | 2.40 (2.32-2.49) | | | 2.46 (2.37-2.54) |
|  | No | 10517 (39.0) | 7917 (21.0) | *Ref* | | | |
| COR = Crude Odds ratio, AOR =Adjusted Odds Ratio, *Ref* = reference category | | | | | | | |
