# Supplementary figures and images for: Disease Characteristics, Care-Seeking Behavior, and Outcomes Associated With the Use of AYUSH-64 in COVID-19 Patients in Home Isolation in India: A Community-Based Cross-Sectional Analysis
Source: Front Public Health. 2022 Jul 6;10:904279. doi: 10.3389/fpubh.2022.904279 (PMC9310753; doi:10.3389/fpubh.2022.904279)

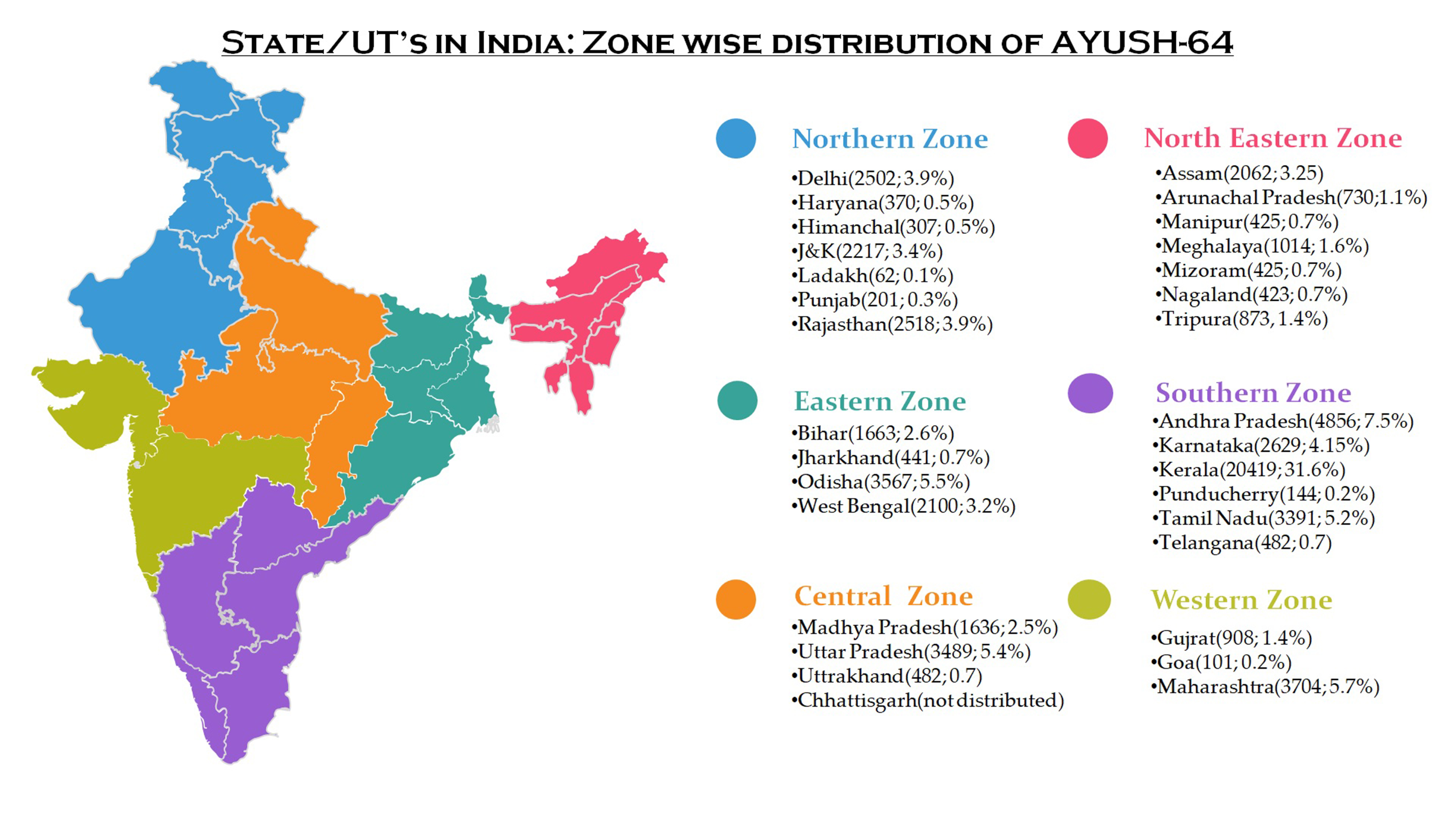

Supplement: Supplementary file 2 [file Image_1.JPEG]
