# Supplementary material for: Disease Characteristics, Care-Seeking Behavior, and Outcomes Associated With the Use of AYUSH-64 in COVID-19 Patients in Home Isolation in India: A Community-Based Cross-Sectional Analysis
Source: Front Public Health. 2022 Jul 6;10:904279. doi: 10.3389/fpubh.2022.904279 (PMC9310753; doi:10.3389/fpubh.2022.904279)
Supplement: Supplementary file 3 [file Data_Sheet_1.doc]

**Physician reporting Format (Day 1)**

1. **Has the patient agreed to take AYUSH-64 and provide the requisite information**

- Yes
- No

1. **Name of the Nodal Officer**
2. **Mobile Number of the Nodal Officer**
3. **State**
4. **Name of the patient**
5. **Age of the patient (18 – 60 years)**
6. **Sex**

- Male
- Female
- Others

1. **Mobile number of the patient / care giver:**
2. **Aadhar Number / Voter Id / Any other government ID of the patient**
3. **Name and relation of the caregiver**
4. **Does the patient belong to any category who are at higher risk of contracting the disease**

- COVID frontline worker
- General health worker
- Occupation requiring frequent social/public interaction
- No direct interaction with public (None of the above)

1. **Marital Status**

- Married
- Unmarried
- Others

1. **Substance Abuse**

- None
- Smoking
- Tobacco Chewing
- Alcohol
- Others

1. **How was the patient identified as having COVID-19?**

a) Positive RT-PCR for COVID-19

b) Positive Rapid antigen test for COVID-19

c) Diagnosed on the basis of COVID-19 symptoms

If a or b is selected then

Date of RT-PCR / Rapid Antigen Test (DD/MM/YYYY) __________

If c) is selected then

Date of onset of Symptoms (DD/MM/YYYY) ___________

1. **Reason for testing:**
2. Chance of exposure
3. Onset of symptoms
4. Random testing (Testing done in health camps, offices, stations, airports)
5. Underwent testing for other reasons
6. Days since the patient started home isolation __________
7. **Vaccination Status:**
8. Fully Vaccinated
9. Single dose vaccinated
10. Not done

If a) or b) from Q. No 23 is selected then

1. Date of single dose/ fully vaccinated (DD/MM/YYYY) ________
2. If vaccinated, name of the vaccine

- COVAXIN
- COVISHIELD
- Don’t Know
- Others: ______________________________

1. **Symptom Status**
   - Asymptomatic
   - Symptomatic

If symptomatic (Mandatory for those who select symptomatic in Q. No 24 above)

a) Severity of disease

- Mild
- Moderate

b) Symptoms in the patient (Mandatory for those who are symptomatic)

Fever

*If fever is present, highest temperature recorded in the last 48 hours (in ^0^F):_________*

Sore throat

Cough

Headache

Body ache

Tiredness

Difficulty in breathing

Loss of smell

Loss of taste

Rhinitis

Insomnia

Anxiety

Diarrhea

Vomiting

Loss of appetite

Abdominal pain

Weakness

1. **Does the patient have any co-morbidities?**

- Yes
- No

If selected yes, then

**Co-morbidities**

- Diabetes
- Hypertension
- COPD
- Asthma
- Cardiovascular diseases
- Immunodeficiency
- Renal Disease
- Liver disease
- Malignancy / History of malignancy

1. **Is the patient taking conventional standard care medicines?**

- Yes
- No

If yes, then, Medicines being taken by patient for COVID 19:

- Methylprednisolone
- Dexamethasone
- Inhalational Budesonide
- Tab Ivermectin
- Tab Paracetamol
- Tab Azithromycin
- T.Vitamin C
- T.Zinc

Other modern medicines for COVID 19: ___________

1. Any other medicines that is being taken by the patient (AYUSH medicines/Home remedies etc):_________________________________
2. Medicine prescribed

- AYUSH-64 (2 tablets twice daily for 20 days) (For Asymptomatic patients)
- AYUSH-64 (2 tablets thrice daily for 20 days) (For Symptomatic mild to moderate patients)

1. Remarks _________________________

**Day 21 Evaluation - Physician Reporting Format**

1. Name of the Nodal officer
2. Mobile number of the Nodal Officer
3. State
4. Name of the patient
5. Mobile number of the patient / care giver
6. Aadhar number of the patient / Voter ID / Any other government ID
7. Was the Nodal Officer able to contact the patient for telephonic interview?

- Yes
- No

1. Has the patient tested negative for COVID-19?
2. Yes (Tested Negative)
3. No (Tested Positive)
4. Test result awaited
5. Not tested

If answer to Q. no. 8 above is either a), b) or c), then

I. Type of test

- RT-PCR test
- Rapid Antigen Test

II. Date of latest RT-PCR / Rapid antigen test (DD/MM/YYYY) (Mandatory for all those who select either of the option a), b) or c) to Q.NO 8 above) ________________

1. Did the condition of the patient undergo worsening anytime during the last one week?

- Yes
- No

If the answer to Q. No 9) above is Yes then select the options which are applicable

- Required additional medical consultation for it
- Required oxygen support
- Required hospitalization
- Admitted in an ICU setting
- Required ventilator support

1. Present Status of symptoms

- Asymptomatic
- Symptomatic

If Answer to Q. No 10 above is asymptomatic then

If asymptomatic is selected, then when did the patient become symptom free (Applicable for only those patients who were symptomatic at previous visit) Date (DD/MM/YYYY) ______________________

If answer to Q. no. 10 is symptomatic, symptoms present now

- Fever
- *If fever is present, highest temperature recorded in the last 48 hours ( in ^0^F):_________*
- Sore Throat
- Cough
- Headache
- Body ache
- Tiredness
- Difficulty in breathing
- Loss of smell
- Loss of taste
- Rhinitis
- Insomnia
- Anxiety
- Diarrhea
- Vomiting
- Loss of appetite
- Abdominal pain
- Weakness

1. Which of the following medicines was issued to the patient at baseline?

- AYUSH-64
- Kabasura Kudineer

1. No. of AYUSH-64 tablets remaining____________
2. Reason for non-compliance

- No reason
- Due to onset of AE
- Due to worsening of symptoms
- Not applicable (Using medicines as per the instructions)

1. Did the patient develop any AE during the use of AYUSH 64?

- Yes
- No

If the answer to Q. no.14 above is Yes, then

I. Specify the AE with duration___________________________________________________

II. Do you think there is an association of AE with the dispensed intervention

- Certain
- Probable / likely
- Possible
- Unlikely
- Unclassified
- Cannot be assessed

1. Was there any need of changing the dose of AYUSH-64 prescribed during the previous assessment?

- Changed from 2-0-2 to 2-2-2
- No change

1. Status of the patient

- Completed
- Drop out

1. Remarks ______________________
